# Bridging the Gap: Exploring the Preclinical Potential of *Pereskia grandifolia* in Metabolic-Associated Fatty Liver Disease

**DOI:** 10.1155/2023/8840427

**Published:** 2023-11-09

**Authors:** Edilson Rodrigues Albuquerque, Gustavo Ratti da Silva, Fernanda de Abreu Braga, Ester Pelegrini Silva, Karina Sposito Negrini, Julia Amanda Rodrigues Fracasso, Lucas Pires Guarnier, Ezilda Jacomassi, João Tadeu Ribeiro-Paes, Roberto da Silva Gomes, Arquimedes Gasparotto Junior, Francislaine Aparecida dos Reis Lívero

**Affiliations:** ^1^Laboratory of Preclinical Research of Natural Products, Post Graduate Program in Animal Science with Emphasis on Bioactive Products, Universidade Paranaense, Umuarama, Brazil; ^2^Laboratory of Preclinical Research of Natural Products, Paranaense University, Umuarama, Brazil; ^3^School of Dentistry, São Paulo State University, Araçatuba, Brazil; ^4^Department of Genetic, Ribeirão Preto Medical School, University of São Paulo, Ribeirão Preto, São Paulo, Brazil; ^5^Laboratory of Preclinical Research of Natural Products, Post Graduate Program in Medicinal Plants and Phytotherapeutics in Basic Attention, Paranaense University, Umuarama, Brazil; ^6^Department of Biotechnology, São Paulo State University, Assis, São Paulo, Brazil; ^7^Department of Pharmaceutical Sciences, North Dakota State University, Fargo, North Dakota 58102, USA; ^8^Laboratory of Cardiovascular Pharmacology, Faculty of Health Sciences, Federal University of Grande Dourados, Dourados, Brazil; ^9^Laboratory of Cardiometabolic Pharmacology, Federal University of Paraná, Curitiba, Brazil

## Abstract

Metabolic-associated fatty liver disease (MAFLD) is a complex condition characterized by steatosis and metabolic disturbances. Risk factors such as diabetes, cigarette smoking, and dyslipidaemia contribute to its development and progression. Effective and safe therapies for MAFLD are urgently needed. *Pereskia grandifolia* has shown potential as an alternative treatment, but its effectiveness against liver disease remains unexplored. This research aims to determine the hepatoprotective properties of *P. grandifolia* using a model of MAFLD. The study was carried out through various phases to assess the safety and efficacy of the ethanol-soluble fraction of *P. grandifolia*. Initially, an *in vitro* assay was performed to assess cell viability. This was followed by an acute toxicity test conducted in rats to determine the safety profile of the extract. Subsequently, the anti-inflammatory properties of *P. grandifolia* were examined in macrophages. For the MAFLD study, diabetic Wistar rats were made diabetic and exposed to a high fat diet and cigarette smoke, for 4 weeks. During the last 2 weeks, the rats were orally given either the vehicle (negative control group; C-), *P. grandifolia* (30, 100, and 300 mg/kg), or insulin in addition to simvastatin. A basal group of rats not exposed to these risk factors was also assessed. Blood samples were collected to measure cholesterol, triglycerides, glucose, ALT, and AST levels. Liver was assessed for lipid and oxidative markers, and liver histopathology was examined. *P. grandifolia* showed no signs of toxicity. It demonstrated anti-inflammatory effects by inhibiting phagocytosis and macrophage spreading. The MAFLD model induced liver abnormalities, including increased AST, ALT, disrupted lipid profile, oxidative stress, and significant hepatic damage. However, *P. grandifolia* effectively reversed these changes, highlighting its potential as a therapeutic agent. These findings emphasize the significance of *P. grandifolia* in mitigating hepatic consequences associated with various risk factors.

## 1. Introduction

Metabolic-associated fatty liver disease (MAFLD) is an increasingly prevalent health concern globally [1]. It entails the buildup of fatty deposits within the liver, causing inflammation, fibrosis, and harm to the liver. MAFLD has now become the prevailing chronic liver ailment worldwide and is influenced by a combination of genetic, environmental, and behavioral factors [2]. Obesity, insulin resistance, type 2 diabetes, dyslipidaemia, tabagism, and sedentary lifestyle are some of the risk factors associated with MAFLD. Therefore, effectively tackling this condition necessitates a comprehensive approach that considers all these aspects [3].

Diabetes mellitus significantly increases the risk of MAFLD development and progression. Insulin resistance, a hallmark of diabetes, promotes hepatic lipid accumulation by altering fatty acid oxidation and increasing de novo lipogenesis. Hyperglycemia and chronic inflammation also contribute to liver injury, fibrosis, and the progression of MAFLD [4, 5]. Cigarette smoking has been recognized as an independent risk element for MAFLD particularly in individuals with diabetes. Smoking-induced oxidative stress and systemic inflammation exacerbate insulin resistance, promote hepatic lipid accumulation, and increase the risk of liver injury and inflammation [6, 7]. Finally, dyslipidaemia, characterized by elevated low density lipoprotein and triglycerides levels, frequently coexists with MAFLD and contributes to hepatic steatosis by increasing lipid delivery to the liver and impairing hepatic lipid metabolism. It promotes inflammation, oxidative stress, and fibrosis, exacerbating MAFLD progression [8].

It is important to note that the combination of diabetes, cigarette smoking, and dyslipidaemia can have a synergistic impact on MAFLD development and progression. These factors interact through various pathways, including oxidative stress, chronic inflammation, impaired lipid metabolism, and insulin resistance, resulting in amplified liver damage and fibrosis. Their combined effects can accelerate disease progression and increase the risk of complications [9, 10]. In this way, understanding the influence of diabetes mellitus, cigarette smoking, and dyslipidaemia on MAFLD is essential for comprehensive disease management strategies.

One of the main challenges in the treatment of MAFLD is its multifactorial nature, and unfortunately, there is no specific approved pharmacological treatment for the condition. Given the interplay between diabetes, cigarette smoking, dyslipidaemia, and MAFLD, comprehensive management strategies are crucial. Multidisciplinary approaches that target glycemic control, smoking cessation, and dyslipidaemia management are essential in MAFLD treatment. However, the drugs currently available for the treatment of the disease have limited efficacy and exert significant adverse effects [11].

In this line, animal models with multiple risk factors associated with MAFLD can provide valuable information on the complex pathogenesis of the disease and potential therapeutic targets. The proposition of these animal models is justified by the need to address the complex nature of MAFLD and the limited effectiveness of current treatments [12–15]. By utilizing these models, it is possible to enhance the likelihood of identifying promising therapeutic candidates for further development and clinical trials [16].

Under these circumstances, medicinal plants are acknowledged as a valuable reservoir for the exploration and advancement of novel pharmaceuticals. For centuries, traditional communities have taken advantage of the therapeutic properties of various plant species to address a broad spectrum of illnesses. Today, scientific research is shedding light on the vast potential of medicinal plants as a valuable reservoir of phytochemicals constituents. Medicinal plants offer a vast repertoire of bioactive compounds that have the potential to act as a reservoir for the identification of novel drugs [17].

*Pereskia grandifolia* Haw. (Cactaceae), commonly known as “ora-pro-nóbis,” has a variety of medicinal properties that have been recognised and utilized for centuries. This tropical plant is a valuable resource in traditional medicine due to its numerous therapeutic benefits. Its leaves are traditionally used for dietary supplementation (due to their rich protein content) and for their antioxidant, antitumor, anti-inflammatory, lipid-lowering, antidiabetic, and hypotensive effects [18–22]. However, pharmacological research into its effectiveness in treating liver disorders remains lacking. Considering the extensive traditional use of *P. grandifolia* for addressing liver conditions and its promising medicinal properties, it is plausible to explore this species as a potential hepatoprotective agent, particularly for liver diseases associated with multiple risk factors. Hence, this study aims to establish a preclinical model of MAFLD incorporating dyslipidaemia, diabetes, and smoking, with the objective of assessing the hepatoprotective actions of *P. grandifolia*.

## 2. Materials and Methods

### 2.1. Preparation of *Pereskia grandifolia* Extract

The leaves of *P. grandifolia* were gathered in June 2021 at the Medicinal Plants Garden of Paranaense University (UNIPAR), situated at an elevation of 430 meters above sea level (S23°46′11.3″–W53°16′412″). A voucher sample (number 142) was archived in the UNIPAR herbarium. The plant underwent a drying process in an oven and subsequent spraying. The infusion extract was prepared using the approach detailed in Barbosa et al. [12]. The crushed leaves (100 g) were utilized for the infusion extraction, employing 1 L of boiling water. The resultant infusion was stored in an amber flask for 5 hours. Following filtration, 95% ethanol was introduced to the infusion at a 1 : 3 (v/v) ratio to precipitate proteins and polysaccharides, resulting in a separated heterogeneous phase, which was then filtered out. The ethanol-soluble fraction underwent concentration using a rotary evaporator and subsequent lyophilization. The accuracy of the plant name was verified on https://www.theplantlist.org and was confirmed to be accurate.

### 2.2. Toxicological Studies

#### 2.2.1. *In Vitro* Cytotoxicity of *Pereskia grandifolia*

The 3-(4,5-dimethylthiazol-2,5-diphenyl) tetrazolium bromide (MTT) cytotoxicity assay was conducted according to the method previously described by Tsuboy et al. [23]. Mouse dermal fibroblasts (NIH/3T3, ATCC® CRL-1658™) and hepatocellular carcinoma HUH7 cells were seeded into 96-well microtiter plates and incubated in culture medium for 24 hours at 37°C with 5% CO_2_. When the cells reached approximately 75% confluence after 24 hours, they were exposed to five different concentrations of *P. grandifolia* (100, 200, 400, 800, and 1,600 *μ*g/mL). For the negative control (NC), the extract was substituted with a physiological solution, while for the positive control (PC), it was replaced with 2% (v/v) Tween 80. The treatments were conducted for 24, 48, and 72 hours.

#### 2.2.2. Acute Toxicity Evaluation of *Pereskia grandifolia*

*(1) Animals*. The toxic impacts of the *P. grandifolia* extract were investigated in adult male Wistar rats weighing between 300 and 400 g, sourced from Maringá State University. These rats were accommodated in the vivarium of the laboratory for preclinical research of UNIPAR, with unrestricted access to both liquid and solid diets, while maintaining precise environmental conditions (temperature: 22°C ± 2°C; relative humidity: 50% ± 10%; and 12-hour light/dark cycle). Ethical clearance for the animal study protocols was granted by the Ethics Committee in Research involving Animal Experimentation of UNIPAR (protocol no. SPP2020031000167). The animals were provided with environmental enrichment to enhance their overall well-being. All guidelines and recommendations pertaining to animal welfare, including minimizing the number of animals used and transparent reporting of animal research, were diligently followed [24].

*(2) Experimental Design*. The assessment of acute toxicity followed the guidelines outlined by the Organization for Economic Cooperation and Development (OECD) guideline 423. The initial dose administered was 300 mg/kg due to the absence of prior information on *P. grandifolia* extract. Subsequent animal groups received either higher or lower fixed doses based on the presence or absence of signs of toxicity or mortality. This iterative process continued until the dose causing evident toxicity was identified or no more than one death occurred, when no effects were observed at the highest dose, or when deaths were noted at the lowest dose. The *P. grandifolia* extract was administered orally via gavage in a single dose after an 8-hour fast. Following treatment, the rats underwent an additional 4-hour fasting period. A control group of animals (*n* = 6), treated with the vehicle (filtered water), was included for comparison.

*(3) Assessment of Clinical Signs*. After the administration of *P. grandifolia*, the animals were carefully observed at designated time intervals, which included the initial 30 minutes, as well as 1, 2, 3, and 4 hours post-treatment. A range of behavioral parameters and clinical signs were recorded, encompassing self-grooming, piloerection, labored breathing (dyspnea), abdominal constriction, diarrhea, weakness (prostration), lack of coordination (ataxia), drowsiness (sedation), unconsciousness (coma), and mortality. After the initial 4-hour observation period, the animals were provided with water and food and observed daily for a total of 14 days to document any clinical changes and occurrences of mortality, according to proposed by Silva et al. [25].

*(4) Euthanasia, Material Collection, and Analysis.* On the 15th day, following a 12-hour fasting period, the rats were euthanized using deep anesthesia with isoflurane in a saturation chamber (maintaining a concentration of 1–3%). Blood samples were collected via decapitation for subsequent analysis. Levels of cholesterol, triglycerides, aspartate aminotransferase, alanine aminotransferase, urea, and creatinine were measured using commercially available kits within an automated system. Several blood parameters were recorded, encompassing red blood cell counts, hemoglobin levels, hematocrit, mean corpuscular volume, mean corpuscular hemoglobin, mean corpuscular hemoglobin concentration, leukocyte counts, rod-shaped cells, lymphocytes, and platelet counts. Furthermore, the liver, spleen, heart, and kidneys were meticulously dissected and weighed using an analytical balance. Regarding kidney mass, the average mass between the right and left sides was taken into account. To calculate the relative organ mass (%), the mass of each organ was multiplied by 100 and divided by the body weight of the animal before euthanasia. Small sections of the liver, spleen, heart, kidney, and brain were carefully excised, rinsed with chilled saline solution, and then preserved in 10% buffered formalin. Following established histological procedures, the tissue sections were stained with hematoxylin and eosin and subsequently examined by a veterinarian pathologist for histopathological analysis. The slides containing the tissue samples were observed using a Leica DM 2500 optical microscope to identify any cellular alterations resulting from the administered treatments.

### 2.3. Pharmacological Studies

#### 2.3.1. *In Vitro* Anti-Inflammatory Activity of *Pereskia grandifolia*

*(1) Treatments of Macrophage Cells*. In the entirety of the anti-inflammatory experiments, the dry extract from *P. grandifolia* underwent dissolution in distilled water at the subsequent concentrations: 200 *μ*g/mL, 400 *μ*g/mL, and 600 *μ*g/mL. Within the positive control (PC) group, the extract doses were replaced with dexamethasone (40 *μ*g/mL), whereas the negative control (NC) group received a substitution of a saline solution.

*(2) Culture and Selection of Macrophages*. Raw 264.7 murine macrophages of the ATCC TIB-71 strain were thawed and cultured in a cell culture flask using Dulbecco's modified Eagle medium (DMEM) Ham's F-12 culture medium, maintained at 37°C with 5% CO_2_. The cells were allowed to grow until reached a confluence of 70–80%. Afterward, cell harvesting was performed using a cell scraper, followed by cell counting in a Neubauer chamber and centrifugation at 1500 rpm for 5 minutes. The supernatant was removed, and the cells were resuspended in the culture medium to attain the desired concentration for the assessment of spreading and phagocytosis.

*(3) Macrophage Spreading*. The methodology employed in this study was adapted from Fracasso et al. [26]. Prepared slides were examined using an optical microscope at 400× magnification, with a total count of 100 cells. The calculation of the inhibition of spreading (%) was performed using the following formula: (Inhibition of spreading (%) = (*E*0 − *ET*)/*E*0 × 100). In this equation, *E*0 represents the average number of cells spread in the NC group, while *ET* represents the average number of spread cells in the treated groups.

*(4) Inhibition of Phagocytosis*. Prepared slides were examined using an optical microscope at 400× magnification, and a total of 100 cells were counted, following the protocol proposed by Fracasso et al. [26]. This experiment was conducted in triplicate. The inhibition of phagocytosis (IP) was calculated using the following formula: (IP (%) = (*E*0 − *ET*)/*E*0 × 100). In this formula, *E*0 denotes the average number of cells in the NC group that phagocytosed zymosan particles, and *ET* represents the average number of cells in the treated groups that phagocytosed these particles.

#### 2.3.2. Animals

The research model involved the development of male Wistar rats with a weight range of 200–250 g. These rats were obtained from the central vivarium of the State University of Maringá and then transferred to the vivarium of the laboratory for preclinical research of UNIPAR. They were maintained under carefully regulated environmental conditions, which included a temperature within the range of 22°C ± 2°C, a relative humidity maintained at 50 ± 10%, and a consistent 12-hour light/dark cycle. The rats had free access to food and water and were provided with environmental enrichment. The experiments were conducted during the light phase. Animals were randomly divided into experimental groups, with 8 rats per group. The selection of this group size was based on previous results from similar experimental protocols and adhered to the 3R principles (reduce, refine, and replace) [12–14, 27–29]. Weights were assessed on a weekly basis using an analytical balance. The experimental plan obtained clearance from the Ethics Committee on Animal Use at Paranaense University, identified by protocol number SPP2020031000167. All applicable national and international guidelines were strictly adhered to throughout the study. The animal research findings were reported and analyzed in compliance with the Animal Research Reporting of in vivo Experiments (ARRIVE) guidelines [24].

#### 2.3.3. Experimental Design and Treatments

**Figure 1:**
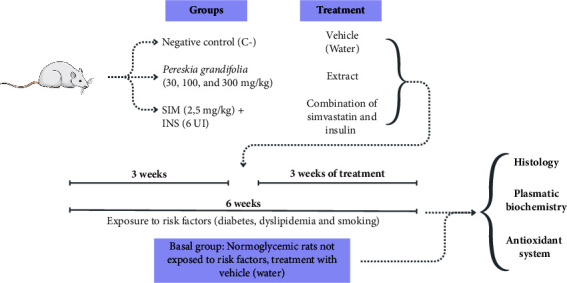
Experimental design: the model of fatty liver disease associated with metabolism was induced with three risk factors: diabetes, dyslipidemia, and smoking. The therapeutic effect of *Pereskia grandifolia* extract (30, 100, and 300 mg/kg) and standard treatment with simvastatin + insulin was evaluated. The baseline group of rats not exposed to risk factors was included as the control group.

The experimental model for metabolically associated fatty liver disease (MAFLD) in this study encompassed a combination of various risk factors such as diabetes, dyslipidemia, and tobacco smoking. At the onset of the treatment, the animals were allocated randomly into respective groups. Induction of diabetes was achieved by subjecting the animals to a 12-hour fasting period, succeeded by intraperitoneal injection of streptozotocin (60 mg/kg) diluted in citrate buffer (10 mM, pH 4.5), following the procedure outlined by Souza et al. [27]. Three days postdrug administration, peripheral blood samples were gathered from the lateral tail vein, and blood glucose levels were determined using an AccuCheck Active glucose meter (Roche®, São Paulo, Brazil). Rats exhibiting glycemia levels ≥250 mg/dL were categorized as diabetic. To induce dyslipidemia, the animals were provided with a standard commercial diet enriched with 0.5% cholesterol for a duration of 6 weeks. This diet comprised 150 g of standard rodent feed combined with one egg yolk and 13.5 mL of corn oil. The mixture was moistened with water, baked in an oven at 50°C for 36 hours, and subsequently vacuum-packed. Each 150 g of this diet contained 225 mg of cholesterol, 1.8 g of saturated fat, 2.16 g of monounsaturated fatty acids, and 0.72 g of polyunsaturated fatty acids. In addition, the rats were exposed to the smoke from nine commercial cigarettes (containing 0.8 mg nicotine, 10 mg tar, and 10 mg carbon monoxide) for 1 hour daily, 5 days a week. Diabetic animals were subjected to the specified risk factors (dyslipidemia and smoking) for a period of 6 weeks, following the protocol proposed by Souza et al. [27]. In the final 3 weeks of the experiment, the animals received daily oral treatment via gavage with either the vehicle (filtered water, serving as the negative control (C-) group), *P. grandifolia* extract (at doses of 30, 100, and 300 mg/kg), or a combination of simvastatin (2.5 mg/kg) and insulin (6 IU, administered subcutaneously; SIM + INS group). Concurrently, a baseline group comprising of normoglycemic, nondyslipidemic, and nonsmoke-exposed rats was treated solely with the vehicle. The experimental setup is depicted in Figure 1.

#### 2.3.4. Euthanasia, Sample Collection, and Biochemical Analysis

At the conclusion of the four-week experimental period, subsequent to a 12-hour fasting period, the animals were humanely euthanized through the administration of deep isoflurane anesthesia within a saturation chamber (20%). Blood samples were obtained through decapitation, and plasma was isolated by centrifugation at 1,500 × *g* for 10 minutes, then stored at −80°C for subsequent analysis of alanine aminotransferase (ALT), aspartate aminotransferase (AST), cholesterol, and triglycerides utilizing the colorimetric enzymatic method on an automated analyzer (Quick Lab, Drake®, São Paulo, Brazil). The liver was then collected, weighed using an analytical balance, and promptly sectioned into samples. One of these samples was rapidly separated and frozen in liquid nitrogen to evaluate oxidative stress and conduct biochemical analyses. Another sample was preserved by submersion in a 10% formalin solution, intended for subsequent histological analysis. In addition, feces accumulated over a two-day period were directly gathered from the animal cages on the final day of the experiment. These fecal samples were preserved at −20°C until processing for the assessment of cholesterol and triglyceride levels.

#### 2.3.5. Determination of Hepatic and Fecal Cholesterol and Triglyceride Levels

The freeze-dried liver and fecal specimens underwent lipid extraction using the gravimetric method outlined by Lívero et al. [30]. In this approach, liver and fecal samples were combined with hexane, serving as the solvent, at a ratio of 1 : 10 (feces: solvent) and were heated to 80°C. Following a 12-hour period, the resulting supernatant was transferred to a second flask and allowed to evaporate naturally. This extraction process was repeated three times. The resulting lipid content was then measured and suspended in a mixture of 1 mL chloroform and 2 mL isopropanol. The levels of triglycerides and cholesterol in both liver and fecal samples were determined using the colorimetric enzymatic technique in an automated analyzer. The lipid percentage in the liver was computed using the formula: (lipids [%] = 100 × [final flask weight/initial flask weight]/0.1 g).

#### 2.3.6. Examination of Hepatic Antioxidant System

To explore the hepatic antioxidant system, the liver samples were initially homogenized using a 1 : 10 dilution of potassium phosphate buffer (0.1 M, pH 6.5). Subsequently, 100 *μ*L of the homogenate was isolated and combined with 80 *μ*L of trichloroacetic acid (12.5%). The resulting mixture was vortexed and then centrifuged at 6,000 rotations per minute (rpm) for 15 minutes at 4°C to assess the levels of reduced glutathione (GSH), following the protocol outlined by Sedlak and Lindsay [31]. The residual homogenate underwent another centrifugation at 9,700 rpm for 20 minutes at 4°C. This additional step was designed to ascertain the superoxide dismutase (SOD) activity and lipoperoxidation (LPO) levels, based on the methodologies established by Gao et al. [32] and Jiang et al. [33], respectively.

#### 2.3.7. Hepatic Histopathological Study

A section of the liver sample was preserved through immersion in a buffered 10% formalin solution, comprising distilled water, 35–40% formaldehyde, and monobasic and dibasic sodium phosphate. Postfixation, the liver sample underwent a dehydration process using alcohol and xylene, and subsequently, it was embedded in paraffin for ease of handling and sectioning. The sections were cut at a thickness of 6 *μ*m and stained with hematoxylin and eosin to visualize cellular structures and any alterations [13]. Another liver sample followed a distinct procedure: it was soaked in sucrose solutions at increasing concentrations (10%, 20%, and 30%) for 24 hours at each level. Following saturation, the liver sample was stored in a medium known as Tissue Tek (O.C.T. Sakura) and quickly frozen using liquid nitrogen. The frozen sample was then sectioned at a thickness of 6 *μ*m and underwent Nile Blue staining, as detailed in the study by Lívero et al. [30]. The stained slides were examined using an optical microscope (Leica DM 2500) to assess cellular abnormalities. The observed hepatic lesions were categorized on a scale from 0 to 3 based on the percentage of affected tissue: 0 (0%; no lesions), 0.5 (1–5%; minor lesions), 1 (6–33%; moderate lesions), 2 (34–66%; marked lesions), and 3 (67–100%; extensive lesions) as per the classification by Mendes et al. [13].

### 2.4. Statistical Analysis

The collected data underwent evaluation for homogeneity of variance and adherence to normal distribution. For comparing means across different groups, a one-way analysis of variance (ANOVA) was executed. In instances where the data did not meet the assumptions of normality or homogeneity of variance, the Kruskal–Wallis test was employed as an alternative. Following this, post hoc tests, such as the Newman–Keuls or Dunnett post hoc test, were utilized to discern specific differences among the means. The threshold for statistical significance was set at 95% confidence (*p* < 0.05). Results are presented as the mean ± standard error of the mean (SEM). The statistical analysis was conducted using GraphPad Prism 9.0 software.

## 3. Results

### 3.1. Impact of *Pereskia grandifolia* on Fibroblast Cell Viability

Table 1 presents the outcomes of an MTT cytotoxicity assay conducted on NIH/3T3 mouse dermal fibroblast cells, aiming to assess the impact of *P. grandifolia* on cell viability at different concentrations and time intervals. The groups treated with varying concentrations of *P. grandifolia* exhibited diverse effects on cell viability. Notably, at a concentration of 100 *μ*g/mL of *P. grandifolia*, the cell viability remained relatively high. These results suggest that *P. grandifolia* at this concentration did not induce a significant cytotoxic effect on the NIH/3T3 cell lineage. At higher concentrations of *P. grandifolia* (200 *μ*g/mL and 400 *μ*g/mL), the cell viability exhibited a noticeable decrease. Moreover, at 400 *μ*g/mL, the viability further declined at 24, 48, and 72 hours. These results suggest that higher concentrations of *P. grandifolia* had a moderate cytotoxic effect on the NIH/3T3 cells. Interestingly, at 800 *μ*g/mL of *P. grandifolia*, the cell viability exhibited a significant decrease at 24 and 48 hours. However, the viability then increased at 72 hours. This indicates that *P. grandifolia* at this particular concentration may have initially induced cytotoxicity, but some cells were able to recover or adapt over time. At the highest concentration of *P. grandifolia* tested (1600 *μ*g/mL), the cell viability was significantly reduced, indicating a strong cytotoxic effect of *P. grandifolia* on the NIH/3T3 cells. The statistical analysis conducted demonstrated that all treatment groups, except for the negative control (NC) group, exhibited significant differences compared to the negative control. The IC50% for *P. grandifolia* in NIH/3T3 cells lineage was determined to be 598.6 *μ*g/mL, 567 *μ*g/mL, and 540.4 *μ*g/mL at 24, 48, and 72 hours. The IC50% values decrease over time, suggesting that the inhibitory potency of *P. grandifolia* increases with longer exposure periods.

### 3.2. Impact of *Pereskia grandifolia* on Carcinoma Cell Type

Table 2 depict the results of an MTT cytotoxicity assay conducted on HUH7 cells to assess the impact of *P. grandifolia* on cell viability. The experiment examined different concentrations of the extract and measured viability at various time points in a carcinoma cell type. The treated groups exhibited varying effects on cell viability when compared to both the negative control (NC) and positive control (PC) groups. At a concentration of 100 *μ*g/mL of *P. grandifolia*, cell viability decreased. These findings indicate a cytotoxic effect of *P. grandifolia* on HUH7 cells at this concentration. Likewise, at concentrations of 200 *μ*g/mL and 400 *μ*g/mL, cell viability continued to decrease. At 200 *μ*g/mL, viability was measured at 41.79 ± 0.09%, 37.61 ± 0.11%, and 17.74 ± 0.28% at 24, 48, and 72 hours. Similarly, at 400 *μ*g/mL, viability was determined to be 35.99 ± 0.08%, 35.35 ± 0.22%, and 15.62 ± 0.41% at the same time intervals. These results suggest a dose-dependent cytotoxic effect of *P. grandifolia* on HUH7 cells. At a concentration of 800 *μ*g/mL of *P. grandifolia*, an interesting observation was made as cell viability increased. This suggests that *P. grandifolia* may have had a different effect on HUH7 cells at this concentration, potentially promoting cell growth or survival. Lastly, at the highest tested concentration of the extract (1600 *μ*g/mL), cell viability decreased once again, indicating a significant cytotoxic effect of *P. grandifolia* on HUH7 cells.

The IC50% value of 183.3 *µ*g/mL at 72 hours indicates that a lower concentration of *P. grandifolia* is required to achieve the desired inhibitory effect compared to the values at 24 and 48 hours. This suggests that longer treatment durations result in increased potency of *P. grandifolia* in inhibiting HUH7 cell growth or viability.

### 3.3. *Pereskia grandifolia* Exhibit Anti-Inflammatory Effects

**Figure 2:**
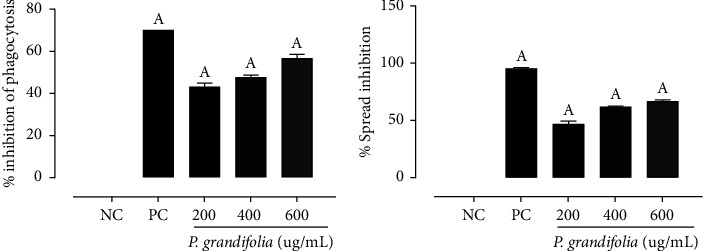
Percentage inhibition of phagocytosis (a) and inhibition of spreading (b) for negative control (NC; physiologic solution 0.9%), positive control (PC; 40 *μ*g/mL, dexamethasone), and *P. grandifolia* (200 *μ*g/mL, 400 *μ*g/mL, and 600 *μ*g/mL). The data are expressed as mean ± SEM. ^A^*p* < 0.05, *vs*. NC group (one-way ANOVA followed by Dunnett's post hoc test).

In phagocytosis, all treatments had significantly different effects compared to the NC group, which received saline (Figure 2(a)). *P. grandifolia* exhibited inhibitory effects on phagocytosis at concentrations of 200 *µ*g/mL, 400 *µ*g/mL, and 600 *µ*g/mL, reducing it by 43.18 ± 1.6%, 47.66 ± 0.99%, and 56.66 ± 1.80%, respectively. The PC group, represented by dexamethasone, inhibited phagocytosis by 70.0%. Spreading is typically defined as an unsuccessful attempt at phagocytosis. In cases where no substance or microorganism is present to be phagocytosed, spreading refers to the action of a responsive cell that adheres to the slide and produces microvilli [34]. All treatments administered in this assay exhibited significantly distinct effects compared to the NC group (Figure 2(b)). *P. grandifolia* exhibited the potential to inhibit spreading, manifesting in the following proportions: 46.99 ± 2.40% at 200 *µ*g/mL, 62.05 ± 1.6% at 400 *µ*g/mL, and 66.87 ± 1.06% at 600 *µ*g/mL. This assay substantiates the findings of the phagocytosis assay, suggesting that *P. grandifolia* holds promise in alleviating inflammation symptoms in macrophages. This potential is comparable to that of 40 *µ*g/mL dexamethasone, which reduced spreading by 70.00%.

### 3.4. *Pereskia grandifolia* Does Not Induce Any Toxic Effects in Rats

During the animal acute toxicity assessment, administering oral doses of *P. grandifolia* did not lead to significant alterations. Specifically, the rats subjected to oral doses of 300 and 2000 mg/kg of *P. grandifolia* did not display any discernible modifications concerning clinical signs, body weight, or relative mass of essential organs such as the liver, spleen, kidneys, or heart, in comparison to the control group. Moreover, there were no notable changes in the biochemical and hematological profiles, and no cellular alterations were identified in the liver, spleen, heart, or kidneys when comparing the experimental groups to the control group (data were not shown).

### 3.5. *Pereskia grandifolia* Did Not Exhibit a Hypoglycemic Effect But It Demonstrated Significant Hepatoprotective Properties

**Figure 3:**
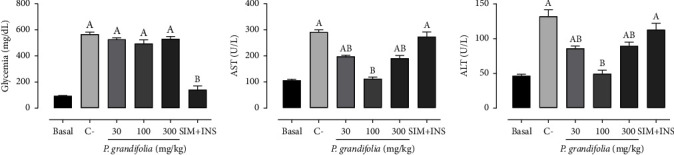
Levels of glucose (mg/dL), hepatic damage biomarkers aspartate aminotransferase (AST; U/L), and alanine aminotransferase (ALT; U/L) in plasma ((a–c), respectively) were measured in normoglycemic, nondyslipidemic, and nonsmoker Wistar rats (basal group) as well as diabetic, dyslipidemic, and smoker Wistar rats treated with vehicle (negative control [C−]), *Pereskia grandifolia* (30, 100, and 300 mg/kg), or simvastatin + insulin (SIM + INS). The presented data represent mean ± SEM. ^A^*p* < 0.05, *vs.* basal; ^B^*p* < 0.05, *vs.* C− (one-way ANOVA followed by Newman–Keuls post hoc test). Glucose and hepatic damage biomarkers levels. Plasma values of (a) glucose (mg/dL), (b) aspartate aminotransferase (AST; U/L), and (c) alanine aminotransferase (ALT; U/L).

Following the induction of diabetes, a significant rise in blood glucose levels was observed in the C-group (566.9 ± 16.5 mg/dL) compared to the basal group (93.3 ± 3.1 mg/dL). Administration of SIM + INS resulted in a decrease in blood glucose levels (141.0 ± 29.1 mg/dL), whereas treatment with *P. grandifolia* partially reverse these alterations (Figure 3(a)). Diabetes, dyslipidaemia, and smoking resulted in a significant rise of approximately 185.1% in ALT and 174.7% in AST levels compared to the basal group (46.3 ± 2.0 U/L and 105.7 ± 4.3 U/L, respectively). Nonetheless, administering 100 mg/kg of *P. grandifolia* extract entirely normalized the heightened levels of ALT and AST. Treatment with 30 and 300 mg/kg *P. grandifolia* extract partially restored these changes, while the combination of SIM + INS showed no significant effect (Figures 3(b) and 3(c)).

### 3.6. *Pereskia grandifolia* Extract Exerted Lipid-Lowering Effects

**Figure 4:**
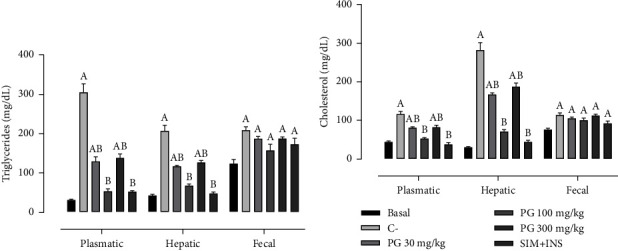
Levels of triglycerides and cholesterol in plasma, liver, and feces ((a and b) respectively) were assessed in normoglycemic, nondyslipidemic, and nonsmoker Wistar rats (basal group) and diabetic, dyslipidemic, and smoker Wistar rats subjected to treatment with vehicle (negative control [C–]), *Pereskia grandifolia* (30, 100, and 300 mg/kg), and simvastatin + insulin (SIM + INS). The study involved *n* = 8 rats per group. The data are expressed as mean ± SEM. ^A^*p* < 0.05, *vs*. basal; ^B^*p* < 0.05, *vs.* C− (one-way ANOVA followed by Newman–Keuls post hoc test). Plasma, hepatic, and fecal levels of (a) triglycerides and (b) cholesterol.

Figure 4 illustrates the effects of *P. grandifolia* treatment on triglyceride and cholesterol levels in the plasma, liver, and feces. The presence of diabetes, dyslipidaemia, and smoking significantly increased plasmatic levels of triglycerides (304.9 ± 21.6 mg/dL) and cholesterol (117.0 ± 6.4 mg/dL) than in the basal group (30.4 ± 1.9 mg/dL and 44.1 ± 2.3 mg/dL, respectively). These risk factors also induced a significant increase in hepatic triglyceride (833.9%) and cholesterol (394.9%) levels compared to that of the basal group (30.3 ± 1.4 mg/dL and 41.8 ± 2.6 mg/dL, respectively). However, administering 100 mg/kg of *P. grandifolia* extract and SIM + INS completely normalized these alterations in both the plasma and liver. Treatment with 30 and 300 mg/kg of *P. grandifolia* extract partially reinstated triglyceride and cholesterol levels in both the plasma and liver. Regarding fecal lipids, the negative control group exhibited a statistically significant increase in fecal cholesterol (75.93 ± 3.3 mg/dL) and triglyceride (113.7 ± 5.8 mg/dL) levels than in the basal group (75.9 ± 3.3 mg/dL and 123.3 ± 10.6 mg/dL, respectively). Interestingly, none of the treatments utilized in this study exhibited the capability to counteract the elevated levels of fecal cholesterol observed in the negative control group.

### 3.7. *Pereskia grandifolia* Reversed Hepatocellular Alterations

**Figure 5:**
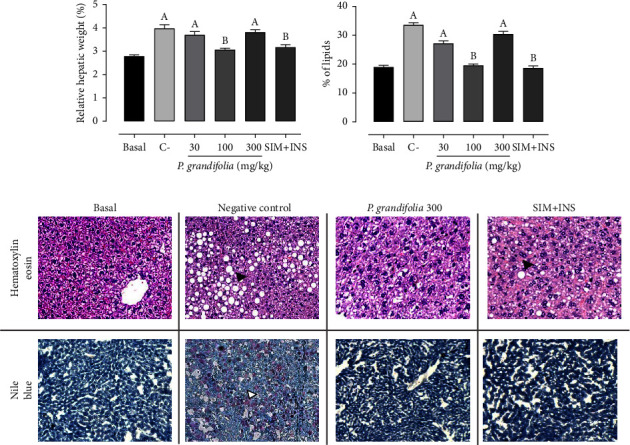
Relative (%) hepatic weight (a), % of hepatic lipids (b), and hepatic histopathological analysis with hematoxylin/eosin and Nile Blue (c) are presented in the figure. The study encompasses two groups: normoglycemic, nondyslipidemic, and nonsmoker Wistar rats (referred to as the basal group) and diabetic, dyslipidemic, and smoker Wistar rats. The latter group was subjected to treatments with vehicle (considered the negative control [C–]), *Pereskia grandifolia* (administered at doses of 30, 100, and 300 mg/kg), and simvastatin + insulin (referred to as SIM + INS). The data are expressed as mean ± SEM. ^A^*p* < 0.05, *vs*. basal; ^B^*p* < 0.05, *vs*. C− (one-way ANOVA followed by Newman–Keuls post hoc test). In the histopathological analysis, hepatic fatty degeneration (steatosis) is indicated by black closed arrows, while triglycerides are indicated by white closed arrows. The magnification used for the images is 20x.

The results depicted in Figure 4 reveal several important findings regarding liver weight, lipid percentage, and histopathological analyses. First, the relative liver weight in the C− group exhibited a notable increase (3.9% ± 0.1%) than that in the basal group (2.7% ± 0.1%). However, the administration of 100 mg/kg *P. grandifolia* extract and SIM + INS effectively normalized this parameter (Figure 5(a)). In addition, the risk factors notably increased the percentage of hepatic lipids by 75.96% in the C− group than that in the basal group (18.9% ± 0.6%). On the contrary, treatment with 100 mg/kg of *P. grandifolia* extract and SIM + INS successfully reversed these changes (Figure 5(b)). Hematoxylin/eosin staining of liver samples confirmed the presence of steatosis, revealing macroscopic alterations and indicating successful induction of hepatic injury (Figure 5(c)). Moreover, Nile Blue staining (Figure 5(c)), which highlights triglycerides in pink, verified the accumulation of lipids. All three risk factors caused cellular alterations, characterized by steatosis. Conversely, no microscopic changes were observed in the liver of the basal group. The combination of the three risk factors resulted in pronounced hepatic steatosis, yielding a lesion score of 3 in vehicle-treated animals. However, treatment with SIM + INS and 100 mg/kg of *P. grandifolia* extract significantly reduced lipid droplets, resulting in a lesion score of 0.5. Furthermore, treatment with 100 mg/kg and 300 mg/kg of *P. grandifolia* extract partially reversed hepatic steatosis, yielding lesion scores of 1 and 2, respectively (data were not shown).

### 3.8. Effects of *Pereskia grandifolia* on Hepatic Redox State

In the rat study, the concurrent presence of diabetes, dyslipidemia, and smoking led to hepatic oxidative stress. These conditions resulted in a decline in the levels of glutathione (GSH), a crucial antioxidant, while simultaneously causing an increase in lipoperoxidation levels when compared to the control group. However, when different doses of *P. grandifolia* were administered, these negative changes were reversed. Notably, treatment with *P. grandifolia* at doses of 30 and 100 mg/kg effectively reduced the heightened activity of SOD observed in the C− group, bringing it closer to the levels seen in the control group. In contrast, treatment with SIM + INS partially restored SOD activity, GSH levels, and lipoperoxidation levels (Table 3).

## 4. Discussion

**Figure 6:**
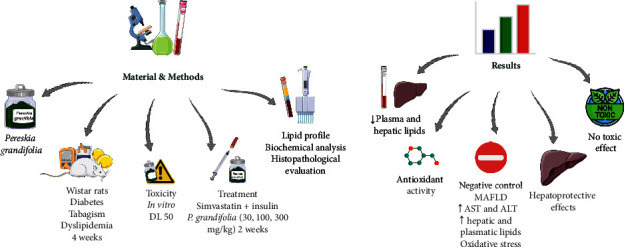
Preclinical ethnopharmacological study examining the impact of *Pereskia grandifolia* on metabolic-associated fatty liver disease (MAFLD) in diabetic rats exposed to a diet high in cholesterol and cigarette smoke. The rats were subjected to various treatments, including vehicle, *P. grandifolia* extract at different doses (30, 100, and 300 mg/kg), or a combination of insulin and simvastatin. The figure showcases the MAFLD model that induced liver abnormalities, such as increased AST and ALT levels, disrupted lipid profile, oxidative stress, and significant liver damage. The results of the study demonstrate the potential of *P. grandifolia* as a promising hepatoprotective agent against MAFLD. The treatment with *P. grandifolia* extract led to improvements in metabolic parameters, lipid levels, and liver histology, without causing any signs of toxicity.

This study explored the hepatoprotective potential of *Pereskia grandifolia* utilizing an experimental model that integrated three significant risk factors for MAFLD: diabetes, dyslipidaemia, and exposure to tobacco smoke. Rats were exposed to these risk factors for a span of 4 weeks, leading to the onset of dyslipidemia, oxidative stress, and evident hepatic anomalies. Nevertheless, daily administration of an ethanol-soluble fraction obtained from *P. grandifolia* leaves effectively reversed these adverse alterations, signifying its promise as a hepatoprotective agent (Figure 6).

Animal models that incorporate multiple risk factors are invaluable for screening new hepatoprotective substances and enable researchers to comprehensively evaluate the efficacy and safety of potential treatments under conditions that closely mirror the complexity of human diseases [35]. By subjecting these models to a combination of risk factors associated with MAFLD, such as diabetes, dyslipidaemia, and tobacco use, it becomes possible to assess the effects of candidate substances on multiple disease mechanisms simultaneously. This multifactorial approach provides a more realistic representation of the disease and enhances our understanding of how potential treatments may interact with various aspects of its pathogenesis.

Moreover, the utilization of animal models that incorporate multiple risk factors enables the identification of synergistic effects of pharmacological treatments [36]. For instance, evaluating the efficacy of a substance in a model encompassing MAFLD, insulin resistance, and other pertinent risk factors allows for the assessment of the combined benefits derived from targeting multiple facets of the disease simultaneously. This approach facilitates an examination of how a drug effectively ameliorates glucose levels and reduces liver fat accumulation within such a model, potentially leading to more pronounced therapeutic benefits compared to substances exclusively focused on a singular aspect of the disease. By comprehensively considering the intricate interplay between diverse disease mechanisms, it is possible to glean valuable insights into potential treatment strategies that optimize therapeutic outcomes for multifactorial diseases such as MAFLD. The results of this study substantiate the promising therapeutic effects of *P. grandifolia* in a model of MAFLD with multiple risk factors. The observed antioxidative, lipid-lowering, and hepatoprotective effects imply that the plant operates through multiple synergistic pathways within the studied model, thus presenting a multifaceted approach to the treatment of MAFLD.

The antioxidant effects of *P. grandifolia* are of great importance in the context of MAFLD, as oxidative stress plays a crucial role in disease progression. Increased levels of reactive oxygen species and lipid peroxide are associated with inflammation and oxidative injury in the liver [37]. The reduction of these markers of oxidative stress following administration of the plant extract suggests its ability to neutralize free radicals and protect liver cells against oxidative damage. Preclinical studies involving multifactorial risk-induced liver disease previously conducted support the results of this research and have highlighted the importance of the antioxidant effect of medicinal plants as a fundamental component in the construction of therapeutic effect [12–14].

The observed hypolipidemic activity is another important characteristic of the therapeutic effects of *P. grandifolia* in MAFLD. The excessive accumulation of lipids, such as triglycerides and cholesterol, in the liver contributes to the development and progression of the disease [8, 38]. The decrease in serum lipid levels, especially when associated with the reduction of hepatic fat accumulation observed in histological analysis, suggests that the plant may modulate lipid metabolism and promote efficient breakdown and transportation of lipids, thereby reducing the lipid burden in the liver. The effect of the plant on reducing hepatic lipids and protecting the liver was previously described by Almeida et al. [22]. Following a 10-week hypercaloric diet, 21 animals were categorized into three groups and provided distinct diets for 4 weeks: control, hypercaloric diet with 5% *P. grandifolia* flour, and hypercaloric diet with 10% *P. grandifolia* flour. Rats subjected to the flour diet exhibited diminished food intake and body weight, as well as reduced body mass and Lee indices in comparison to the control group. In the second week, the 10% *P. grandifolia* flour group demonstrated significantly lower weight compared to the control group. By the fourth week, the 10% *P. grandifolia* flour group experienced the most notable body weight reduction than the other two groups, along with lower hepatic lipid levels and reduced liver weight. However, there was no significant difference in total lipid and moisture levels between the groups [22]. As observed in the referenced study, the rats were exposed to a hypercaloric diet and were provided with feed enriched with 10% *P. grandifolia* for a period of 2 weeks, emphasizing variations in the study design and treatment approach when compared to the current study.

Furthermore, the observed hepatoprotective effects of *P. grandifolia* are particularly encouraging. The decrease in ALT and AST enzyme activities indicates an enhancement in the liver's functional integrity and metabolic functioning, implying a potential safeguard against liver injury [39]. Moreover, *P. grandifolia* demonstrates notable anti-inflammatory activity by inhibiting phagocytosis and macrophage spreading, critical processes in inflammation. Histological analysis further supports these findings, revealing a reduction in liver tissue fat accumulation following treatment with *P. grandifolia* extract. These results suggest that the plant may regulate key metabolic pathways implicated in MAFLD pathogenesis, such as lipogenesis, inflammation, and oxidative stress, thereby providing protection to the liver against damage.

In the study conducted by Barbosa et al. [12], Wistar rats with diabetes were subjected to cigarette smoke exposure and the induction of dyslipidemia for a period of 4 weeks. During the last two weeks of the experiment, the animals received oral treatment with different doses of the ethanol-soluble fraction of *Baccharis trimera* (30, 100, and 300 mg/kg), vehicle (group C−), or a combination of insulin and simvastatin (group C+). This experimental model was designed to induce hepatic steatosis in the animals. Remarkably, the *B. trimera* extract at doses of 30 and 100 mg/kg showed a significant reduction in hepatic lipid levels, including cholesterol and triglycerides, than the animals in the C+ group. Similar results were observed in the group treated with insulin and simvastatin. As for markers of liver injury, diabetic animals with dyslipidemia and cigarette smoke exposure treated with the vehicle showed an increase in AST and ALT levels than that in the C− group. However, all three doses of *B. trimera* demonstrated efficacy in reversing this liver damage. The results suggest that this species holds promise for future studies aimed at the development of a hepatoprotective herbal remedy.

Mendes and colleagues [13] proposed an investigative model associating systemic arterial hypertension, dyslipidemia, and smoking as cardiovascular risk factors. Wistar rats were subjected to a 2K1C Goldblatt hypertension model, received a diet rich in 0.5% cholesterol and were exposed to 9 commercial cigarettes per day for 8 weeks. During the last 4 weeks, the animals received oral treatment with the ethanol-soluble fraction of *Baccharis trimera* (30, 100, and 300 mg/kg) or with a combination of simvastatin (2.5 mg/kg) and enalapril (15 mg/kg). The results revealed that treatment with *B. trimera* was able to reverse all observed alterations, displaying similar or superior effects to the standard treatment, indicating its potential as a promising therapy for cardiovascular and hepatic disorders, especially those associated with multiple risk factors.

In the study conducted by Auth et al. [14], spontaneously hypertensive rats were exposed to a high-cholesterol diet and cigarette smoke for a period of 10 weeks. In the final 5 weeks of this period, the rats were subjected to various treatments including different doses of *Croton urucurana* extract, simvastatin + enalapril, or a vehicle (serving as the negative control). This model induced liver damage, leading to an increase in AST, ALT, cholesterol, and triglyceride levels. Treatment with *C. urucurana* (300 mg/kg) and simvastatin + enalapril effectively mitigated these parameters, successfully reversing the hepatic alterations. These findings underscore the therapeutic potential of *C. urucurana* extract in managing cardiovascular and hepatic conditions associated with multiple risk factors.

Together, the results of this research contribute to the growing evidence supporting the therapeutic potential of medicinal plants in the management of liver diseases, providing valuable insights for the development of treatments and the advancement of hepatic investigations [40–42]. It is important to highlight that the observed therapeutic effects can be attributed to the presence of bioactive compounds in *P. grandifolia*, such as phenolic compounds, flavonoids and saponins, for example [43–45]. However, further research is necessary to identify and characterize the specific compounds present in the extract utilized in this study. In addition, delving into the molecular mechanisms that drive the observed effects is crucial for a more in-depth understanding.

The results of toxicity tests conducted on *P. grandifolia* indicate a high level of tolerance and safety for this plant. Both *in vitro* cell cultures and rat studies have demonstrated its excellent performance. In the cytotoxicity assay, it was observed that the plant had no significant impact on cell viability and proliferation, indicating the absence of direct toxicity on the tested cells. This finding suggests that *P. grandifolia* does not compromise the integrity and functionality of the studied cells, which is a crucial factor to consider in terms of its safety profile. The IC50% values indicate that *P. grandifolia* demonstrates varying inhibitory potencies on cell growth or viability in the two studied cell lines. *P. grandifolia* exhibits a relatively higher potency against HUH7 cells than NIH/3T3 cells. The discovery of *P. grandifolia* selective cytotoxic effect on cancer cells holds great significance in the field of cancer research. Its ability to preserve cell viability in healthy cells shows promising therapeutic potential, as it minimizes the undesirable side effects commonly associated with many conventional therapies. Moreover, the plant cytotoxic effect specifically targeting cancer cells underscores its potential selectivity towards specific molecular targets present in tumor cells.

In the toxicity assay in rats, administration of *P. grandifolia* extract did not result in significant changes in behavior, body weight, or feeding patterns. No lesions or abnormalities were observed in organs and blood biochemical parameters, indicating the absence of systemic toxicity and relevant structural or functional damage in the evaluated tissues. These results suggest that the plant did not cause immediate adverse effects in the studied animals and support the potential therapeutic use of *P. grandifolia*. However, it is important to note that these investigations are an initial step in assessing the safety of the extract. For a comprehensive and more accurate evaluation, further studies are needed, including testing in more diverse animal models, long-term toxicity assessment, and eventually clinical studies in humans [46, 47].

In summary, the results of this study reveal the potential importance of *P. grandifolia* as a hepatoprotective agent in an experimental model of MAFLD. This may have significant implications for public health, medical research, and the appreciation of traditional medicine and culture. MAFLD is an increasingly prevalent liver disease and is associated with risk factors such as diabetes, dyslipidaemia, and smoking [2]. By demonstrating that *P. grandifolia* has hepatoprotective properties, this study may have an impact on public health by providing a possible natural therapeutic approach for the treatment or prevention of MAFLD.

Furthermore, the discovery of the hepatoprotective effects of *P. grandifolia* highlights the potential for further research on this plant and its therapeutic properties. This could lead to the development of drugs or treatments derived from the plant, offering alternatives for addressing liver diseases. In addition, depending on the region, *P. grandifolia* may play a significant role in the traditional medicine of certain communities [48]. This study can provide scientific evidence supporting the traditional use of the plant as a hepatoprotective agent, helping to preserve and promote cultural practices and traditional knowledge related to health.

Although the study provided evidence of the positive impact of *P. grandifolia* treatment on dyslipidaemia, oxidative stress, and hepatic abnormalities, the precise mechanisms responsible for these effects were not fully clarified. To gain a comprehensive understanding, further research is required to unravel the specific molecular targets involved in the observed hepatoprotective effects. Furthermore, while this study investigated the effects of *P. grandifolia*, its clinical relevance and safety in human subjects have yet to be established. To address these aspects, additional studies, including rigorous clinical trials, are necessary. These trials should focus on evaluating the efficacy of *P. grandifolia*, determining the optimal dosage, and assessing potential side effects when administered to humans. Only through such comprehensive investigations will it be possible to gain a clearer understanding of the potential benefits and risks associated with the use of *P. grandifolia* in a clinical setting.

## 5. Conclusion

The experimental model used, which incorporated major risk factors for MAFLD, provided valuable insights into the potential therapeutic benefits of *P. grandifolia* in combating liver damage. This suggests that *P. grandifolia* holds promise as a natural hepatoprotective agent.

## Figures and Tables

**Table 1:** Effect of *Pereskia grandifolia* on fibroblast cell viability: percentage of viability at 24, 48, and 72 hours, and IC_50_% determination.

Treatments	24 h	48 h	72 h
NC	100 ± 0.323	99 ± 0.03	100 ± 0.47
PC	5.94 ± 0.037^*∗*^	4.92 ± 0.097^*∗*^	2.80 ± 0.40^*∗*^
100 *μ*g/mL	91.94 ± 1.59^*∗*^	100 ± 0.758	100 ± 0.157
200 *μ*g/mL	85.91 ± 0.075^*∗*^	73.65 ± 0.517^*∗*^	93.01 ± 0.157^*∗*^
400 *μ*g/mL	74.33 ± 1.02^*∗*^	68.68 ± 0.714^*∗*^	85.37 ± 0.414^*∗*^
800 *μ*g/mL	38.61 ± 3.72^*∗*^	67.83 ± 0.621^*∗*^	20.24 ± 0.031^*∗*^
1600 *μ*g/mL	9.31 ± 0.06^*∗*^	12.31 ± 0.68^*∗*^	5.43 ± 0.36^*∗*^
IC_50_%	598.6 *μ*g/Ml	567 *μ*g/mL	540.4 *μ*g/mL

IC_50_%, half maximal inhibitory concentration; NC, negative control; PC, positive control. The data are expressed as mean ± SEM. ^*∗*^*p* < 0.05, *vs.* NC (one-way ANOVA followed by Dunnett's *post hoc* test).

**Table 2:** Effect of *Pereskia grandifolia* on carcinoma HUH7 cells viability: percentage of viability at 24, 48, and 72 hours, and IC_50_% determination.

Treatments	24 h	48 h	72 h
NC	100 ± 0.17	100 ± 0.22	100 ± 0.22
PC	3.77 ± 0.09^*∗*^	3.15 ± 0.15^*∗*^	3.13 ± 0.221^*∗*^
100 *μ*g/mL	51.58 ± 0.44^*∗*^	41.07 ± 0.08^*∗*^	17.99 ± 0.061^*∗*^
200 *μ*g/mL	41.79 ± 0.09^*∗*^	37.61 ± 0.11^*∗*^	17.74 ± 0.169^*∗*^
400 *μ*g/mL	35.99 ± 0.08^*∗*^	35.35 ± 0.22^*∗*^	15.62 ± 0.23^*∗*^
800 *μ*g/mL	6.01 ± 0.55^*∗*^	13.20 ± 0.27^*∗*^	14.28 ± 0.400^*∗*^
1600 *μ*g/mL	5.83 ± 0.25^*∗*^	10.26 ± 0.17^*∗*^	14.28 ± 0.0.370^*∗*^
IC_50_%	446 *µ*g/Ml	523.4 *µ*g/mL	183.3 *µ*g/mL

IC_50_%, half maximal inhibitory concentration; NC, negative control; PC, positive control. The data are expressed as mean ± SEM. ^*∗*^*p* < 0.05, *vs.* NC (one-way ANOVA followed by Dunnett's *post hoc* test).

**Table 3:** Hepatic markers of oxidative stress in diabetic, dyslipidemic, and smoker Wistar rats that were treated with vehicle, *Pereskia grandifolia*, and simvastatin + insulin.

	Basal	C–	*Pereskia grandifolia* (mg/kg)			SIM + INS
			30	100	300	
GSH	184.50 ± 3.52	29.50 ± 2.11^a^	146.20 ± 7.18^b^	186.20 ± 6.01^b^	151.30 ± 4.11^a,b^	117.60 ± 10.97^a,b^
LPO	63.91 ± 2.74	194.30 ± 3.82^a^	69.13 ± 4.70^b^	57.41 ± 3.22^b^	74.10 ± 2.15^b^	121.40 ± 9.66^a,b^
SOD	1210.0 ± 34.87	1624.2 ± 64.30^a^	1293.0 ± 19.42^b^	1199.0 ± 30.95^b^	1447.5 ± 23.19^a^	1501.8 ± 74.29^a^

C−, negative control; SIM + INS, simvastatin + insulin; GSH, reduced glutathione (*µ*g GSH/g tissue); LPO, lipoperoxidation (nmol LPO/min/mg tissue); SOD, superoxide dismutase (U SOD/mg of tissue). *n* = 8/group. The data are expressed as mean ± SEM. ^a^*p* < 0.05, *vs.* basal; ^b^*p* < 0.05, *vs.* C− (one-way ANOVA followed by Newman-Keuls *post hoc* test).

## Data Availability

The data used to support the findings of this study are available from the corresponding author upon request.
